# Towards an integrated care. A position paper by the Association of the Scientific Medical Societies in Germany (AWMF)

**DOI:** 10.3205/000362

**Published:** 2026-06-18

**Authors:** Steffi G. Riedel-Heller, Manfred Gogol, Thomas Schmitz-Rixen, Vera von Dossow, Rolf-Detlef Treede

**Affiliations:** 1 Department of Social Medicine, Occupational Medicine, and Public Health (ISAP), University of Leipzig Medical Center, Leipzig, Germany; 2 Ad hoc Committee Health Services, Association of the Scientific Medical Societies in Germany (AWMF), Berlin, Germany; 3 Department of Trauma Surgery and Geriatric Traumatology, Hannover Medical School, Hanover, Germany; 4 German Society for Surgery (DGCH), Berlin, Germany; 5 Department of Anesthesiology and Pain Medicine, Heart and Diabetes Center NRW, University Hospitals of the Ruhr-University of Bochum, Medical School OWL (Bielefeld University), Bad Oeynhausen, Germany; 6 Department of Neurophysiology, Mannheim Center for Translational Neuroscience (MCTN), Heidelberg University, Heidelberg, Germany; 7 Department of Psychiatry and Psychotherapy, Central Institute for Mental Health, Medical Faculty Mannheim, Heidelberg University, Mannheim, Germany

**Keywords:** Germany, health care sectors, integrated care, digitisation, remuneration

## Abstract

**Research question:**

Germany’s longstanding separation of healthcare sectors – most prominently between outpatient and inpatient care – creates risks of fragmented service delivery, disrupted information flows, and ultimately suboptimal outcomes for patients. This position paper examines how cross-sectoral and integrated strategies can effectively mitigate or overcome this fragmentation.

**Methods:**

During a Berlin Forum of the Association of the Scientific Medical Societies in Germany (AWMF) (6 December 2024), experts presented best-practice models and discussed legal, structural, financial, and regional dimensions of integrated care. The insights from these discussions were synthesized into AWMF's recommendations.

**Results:**

Integrated care represents a critical lever for improving the efficiency and quality of the German healthcare system. Promising examples exist across surgical, medical, psychiatric, and regionalized care settings. Key recommendations address the following topics: harmonized financing and remuneration system, regional population-based healthcare networks, interoperable information exchange across healthcare sectors, shared decision-making on care options. Health services research – and especially implementation research – plays an indispensable role in guiding and evaluating these reforms. The AWMF further emphasizes the need for integrated education and postgraduate training, particularly within structured residency networks, and offers AWMF as an important interdisciplinary and interprofessional platform for promoting cross-sectoral care.

**Conclusions:**

Broad implementation of integrated care, combined with robust and evidence-based monitoring of implementation, is essential for meeting the challenges posed by demographic change and increasing demands on healthcare delivery.

## Zusammenfassung

**Fragestellung:**

Die bisherige Trennung der Versorgungssektoren in Deutschland – insbesondere zwischen ambulanter und stationärer Versorgung – birgt die Gefahr von unzureichend effizienten Behandlungsabläufen, Informationsverlusten und folglich einer suboptimalen Patientenversorgung. Dieses Positionspapier evaluiert, wie sektorenübergreifende Ansätze diese Fragmentierung verbessern und überwinden können.

**Methoden:**

Im Rahmen eines Berliner Forums der Arbeitsgemeinschaft der Wissenschaftlichen Medizinischen Fachgesellschaften (AWMF) (6. Dezember 2024) wurden Beispiele guter Praxis sowie rechtliche, strukturelle und regionale Aspekte der sektorenübergreifenden Versorgung durch Expertinnen und Experten vorgestellt und diskutiert. Die Ergebnisse flossen in ein Positionspapier ein, das Empfehlungen der AWMF formuliert.

**Ergebnisse:**

Die sektorenübergreifende Versorgung ist ein zentraler Ansatzpunkt zur Verbesserung der Effizienz und Qualität des deutschen Gesundheitssystems. Beispiele guter Praxis sind ermutigend. Die Empfehlungen der AWMF betreffen die folgenden Themenbereiche: Harmonisierung der Finanzierungs- und Vergütungssysteme, Etablierung regionaler Gesundheitsnetzwerke, interoperabler Informationsaustausch über alle Sektoren, partizipative Entscheidungsfindung zu Behandlungsalternativen. Die Versorgungsforschung spielt dabei eine zentrale Rolle. Die AWMF empfiehlt zudem eine sektorenübergreifende Aus- und Weiterbildung z.B. in Weiterbildungsverbünden und bietet die Nutzung der AWMF als strategische Plattform für interdisziplinäre und interprofessionelle Weiterentwicklung sektorenübergreifender Versorgung an.

**Schlussfolgerungen:**

Eine Ausweitung der sektorenübergreifenden Versorgung mit evidenzbasiertem Monitoring der Implementierungsschritte ist essenziell, um den Herausforderungen des demografischen Wandels und den steigenden Anforderungen an die Gesundheitsversorgung zu begegnen.

## 1 Introduction

**Figure 1:**
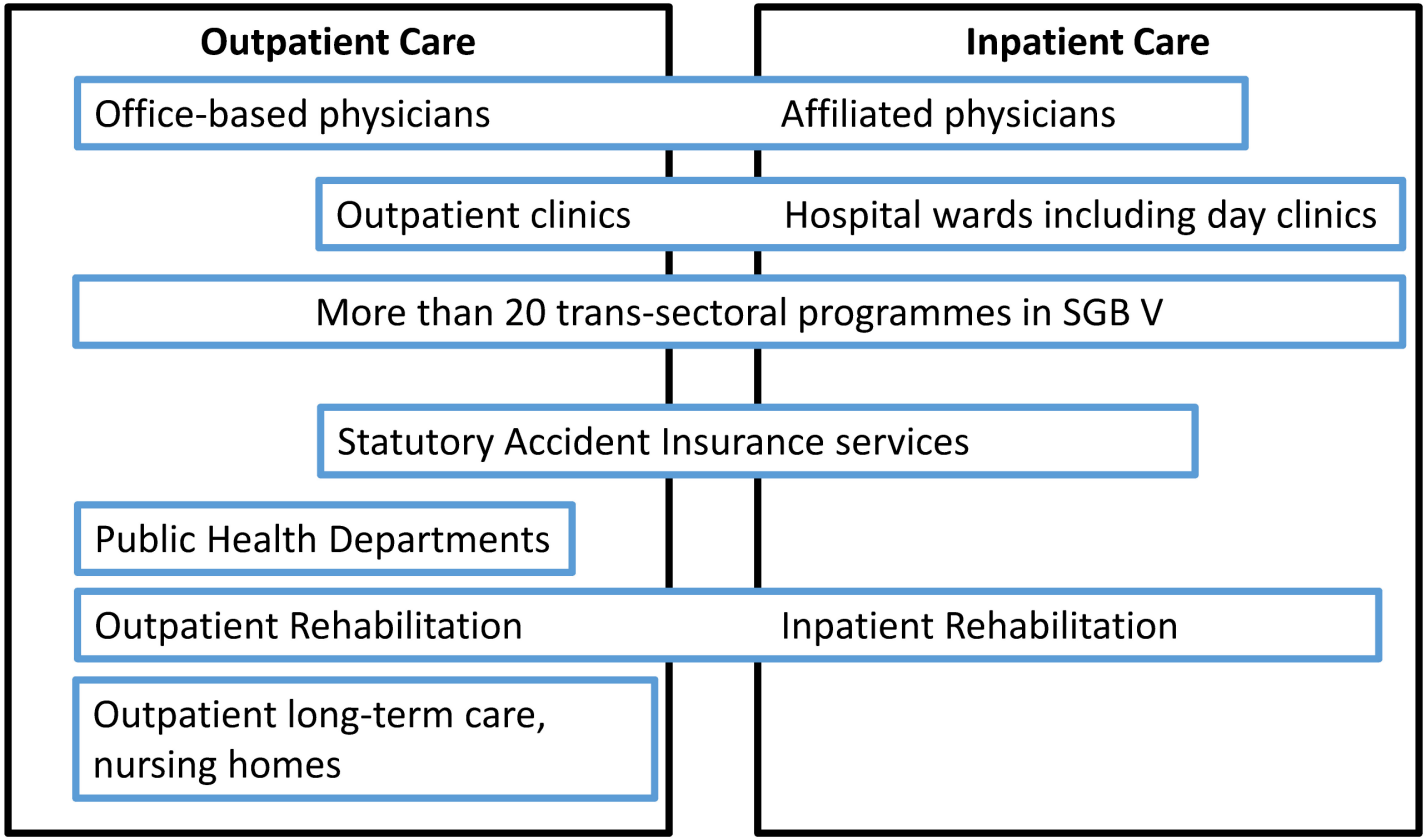
Sectors of the German Healthcare System Organisational chart of the sectors of the German healthcare system, presented for statutory insurance schemes. Sector boundaries arise, among other things, from the various payors: statutory health insurance (GKV) since 1883, statutory accident insurance (GUV) since 1884, statutory pension insurance since 1889, statutory nursing care insurance since 1995. Further boundaries arise from the separate regulation of service providers: doctors, providers of therapeutic services (physiotherapy, speech therapy, etc.), providers of medical aids (medical supply stores). Pharmacies, specialist care. Privately insured persons and self-payers have access to the same service providers, but typically with a uniform payer. Compiled according to [24] and [25].

In 2022, Germany’s healthcare expenditure approached €500 billion – placing the country at the top of European spending levels [1]. Germany also records roughly one-third more outpatient treatments and at least 50% more inpatient treatments than the EU average [2]. Paradoxically, life expectancy tells a different story: at 81.2 years, it fell below the EU average for the first time in 2024, lagging 2.6 to 3 years behind leaders such as Spain, Italy, and Switzerland [3]. This discrepancy raises concern. While the reasons are multifactorial, deficits in prevention and systemic inefficiencies – particularly those stemming from strict sectoral divisions – play a major role. The German healthcare system is characterised by pronounced fragmentation, most visibly in the institutional divide between outpatient care delivered by general practitioners and specialists under the statutory health insurance system and inpatient hospital care (Figure 1 (Fig. 1)). This separation is more rigid in Germany than in most comparable health systems [4]. Additional sectors, such as public health services, rehabilitation, and statutory accident insurance, further contribute to the complexity [2]. Structurally, these divisions reflect the historical evolution of the system: multiple legal frameworks (SGB V, VIII, IX, XI), distinct organisational actors, divergent incentives, and – crucially- separate planning and financing mechanisms.

**Figure 2:**
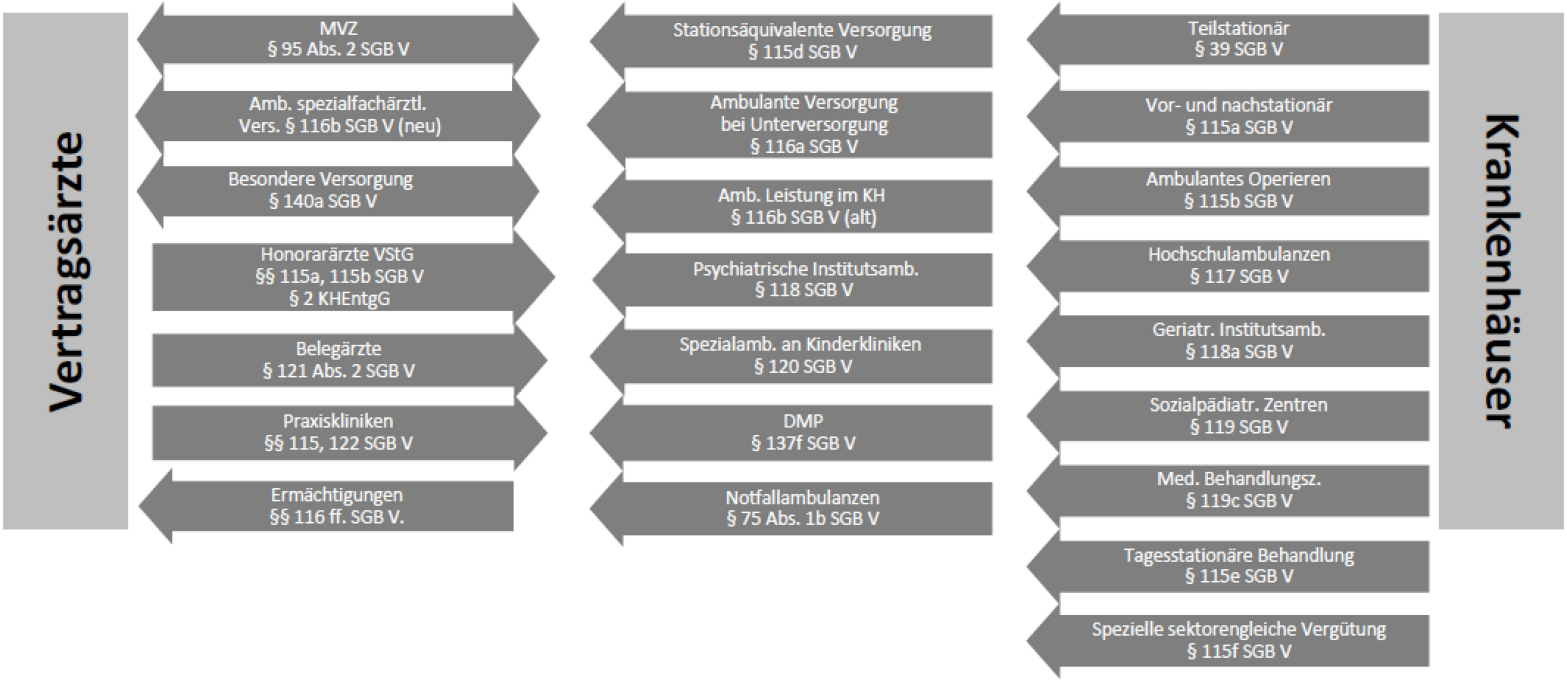
The sectors of the German healthcare system – integrative care approaches There is no shortage of integrative care models, but is their coordination sufficient? Combined outpatient-inpatient regulations in SGB V (overview) [according to Dr Wulf-Dietrich Leber ©, GKV-Spitzenverband; reprinted with kind permission]. SGB V: German social code book V, Vertragsärzte: office-based physicians, Krankenhäuser: hospitals [Figure available in German only]

For patients with chronic or complex conditions, these boundaries can impede seamless care. Although more than 20 integrated programmes exist (Figure 2 (Fig. 2)), most sectors still operate with limited coordination. Transitions, especially between inpatient and outpatient settings, frequently lead to information gaps, inefficiencies, and avoidable costs [5], [6]. These problems are not new. Yet demographic trends will sharply intensify them. Germany faces an ageing population with rising multimorbidity, accompanied by a shrinking workforce responsible for financing and delivering care. This dual pressure heightens the urgency for structural reform. Recognising this, the Association of the Scientific Medical Societies in Germany (AWMF) highlighted the need to improve the quality and coordination of healthcare across all sectors in its position paper for the 2025 federal election [7]. At the AWMF Berlin Forum of December 2024, experts discussed the systemic reorganisation required to move toward a genuinely cross-sectoral and integrated model of care.

## 2 Methods

At the AWMF Berlin Forum held on 6 December 2024, experts from AWMF member societies and key stakeholders across the healthcare system presented best-practice examples illustrating cross-sectoral care in both surgical and medical disciplines (Table 1 (Tab. 1)). These presentations were complemented by discussions of fundamental issues, including the legal and regulatory framework, implications for medical education and postgraduate training, and the specific role of regional structures in enabling integrated care.

The topics introduced in the presentations were subsequently examined in greater depth during a moderated panel discussion. Drawing on this combined input, the AWMF Council synthesised the content into the current position paper. The final text underwent an internal review and consensus process within the Council to ensure accuracy, coherence, and alignment with the strategic priorities of the AWMF.

## 3 Results

### 3.1 Areas in need of improvement

Significant optimisation potential exists at several levels of the German healthcare system, particularly in the areas of intersectoral communication, the standardisation of treatment pathways, and the strengthening of interdisciplinary collaboration. Cross-sectoral care directly addresses these gaps by promoting closer integration of medical specialties and reducing the friction created by rigid sector boundaries. The overarching goal is a genuinely patient-centred care pathway, in which all relevant actors – general practitioners, specialists, hospitals, rehabilitation providers, and public health services – work in a coordinated manner. Cross-sectoral approaches therefore prioritise: integrated treatment pathways, digital infrastructures that support seamless information exchange, regionalisation and population-based planning, and collaborative models that transcend institutional silos. These strategies aim to better reflect the needs of local populations and to make more efficient use of existing healthcare resources.

### 3.2 Making sector boundaries more permeable

Over recent decades, a wide range of initiatives has aimed to make the traditional boundaries between healthcare sectors more permeable. These efforts include legislative reforms, integrated care programmes, and regional pilot projects designed to promote continuity across outpatient and inpatient settings. Integrated care arrangements have been created to link hospitals, office-based physicians, and rehabilitation or long-term care providers, while Medical Care Centres (MVZs) bring multiple specialties together under one organisational roof, often in cooperation with hospitals. Another example is ambulatory specialist care (ASV), which enables multidisciplinary treatment of patients with rare, severe, or complex conditions within a cross-sector legal framework. Digitisation has proven to be one of the strongest enablers of such integration. The introduction of electronic patient records, the development of interoperable IT systems, and the expansion of digital infrastructures provide the foundation for more seamless information flows. Standardised data formats and interfaces are essential to ensure that information can move fluidly between outpatient practices, hospitals, rehabilitation services, and public health authorities. Financial mechanisms also strongly shape the degree of integration. Existing remuneration systems – case-based payment in hospitals versus fee-for-service outpatient billing – create substantial barriers to cooperation. Diagnosis-related groups which align payment for selected procedures across sectors (Hybrid DRGs), represent a promising first step in overcoming these structural divides. In addition, regional and population-based budgeting models offer alternative pathways for fostering coordinated, needs-based care (see Table 2 (Tab. 2)). Regional initiatives such as *Gesundes Kinzigtal* demonstrate how financial alignment, prevention, and coordinated delivery can work together to improve population health outcomes [8]. 

### 3.3 Current developments: The hospital reform

The recommendations of the Government Commission for Modern, Needs-Based Hospital Care (2024) were partially incorporated into the Hospital Care Improvement Act (KHVVG), which was passed by the German Bundestag on 17 October 2024. This legislative step marked the beginning of a far-reaching hospital reform. A central objective of the commission’s work was to address the structural problems arising from the strict separation between outpatient panel physicians and hospital-based care. To this end, the commission proposed a coordinated package of short-, medium-, and long-term measures [2], [9] (Table 3 (Tab. 3)). In addition, it developed a digital planning and impact assessment tool designed to support evidence-informed decision-making and improve transparency in regional capacity planning.

### 3.4 What role do regions play?

The regional level plays a pivotal role in achieving effective and sustainable cross-sector care. Demographic and epidemiological conditions vary greatly across Germany – urban versus rural settings, ageing populations, local workforce shortages, and differing healthcare infrastructures all influence the design and delivery of care. Because of these variations, regional approaches allow for more nuanced, context-sensitive solutions than uniform national regulations. Regions are also critical environments for building networks that link hospitals, office-based physicians, nursing and long-term care providers, social services, and public health authorities. Such networks foster long-term partnerships, the development of shared care cultures, and improved coordination across sectors. Regional actors can respond more rapidly to emerging needs and are better positioned to identify gaps, reallocate resources, and refine care pathways. Moreover, regions provide a valuable testing ground for innovative cross-sector models. Pilot projects can be implemented and evaluated locally, with successful approaches subsequently being scaled to other regions or integrated into national policy. In this way, regional structures become engines of innovation and essential contributors to the development of a more integrated, responsive, and population-oriented healthcare system.

### 3.5 Examples of good practice

#### Inpatient-equivalent treatment (StäB) in psychiatry

Inpatient-equivalent treatment (StäB) provides intensive, home-based psychiatric care for individuals with severe mental illness. A multidisciplinary team delivers time-limited, structured treatment directly in the patient’s home environment, enabling continuity of care and strengthening social and family resources. Internationally, this model has been established for many years and has been positively evaluated in randomised controlled trials, forming part of evidence-based guideline recommendations [10]. The Act on the Further Development of Care and Remuneration for Psychiatric and Psychosomatic Services pursuant to Section 115d of the German Social Code (PsychVVG 2017) established the legal framework for providing such treatment in Germany. German evaluation data from the AKtiV study show that StäB can significantly reduce the need for full inpatient admissions while achieving clinical and functional outcomes comparable to conventional inpatient treatment. Patients and relatives report higher satisfaction, and staff also describe improved working conditions and therapeutic opportunities [11]. However, the nationwide implementation of StäB continues to face several barriers. These include high audit frequencies by the statutory health insurance controlling agency (Medizinischer Dienst), substantial documentation burdens, and often complex negotiations with statutory health insurers [12]. Experts therefore call for regulatory adjustments, particularly greater flexibility in the frequency of home visits and a reduction of administrative obstacles, in order to support broader adoption.

#### Hybrid DRGs as a cross-sector remuneration instrument in surgical care

Hybrid DRGs have been available since 1 January 2024 as a unified reimbursement model for selected surgical procedures listed under §115b SGB V. They are paid irrespective of whether the procedure is performed on an inpatient or outpatient basis and are intended to cover all examinations and treatments directly associated with the intervention. The goal is to remove financial disincentives and encourage the clinically appropriate shift of surgical procedures to the outpatient setting [13]. While the number of procedures included in the hybrid DRG catalogue remains limited, it is gradually expanding. According to initial estimates, the extended catalogue may help avoid approximately 400,000 full inpatient cases from 2025 onward [13]. Early practical experience highlights important areas for refinement. Criticisms include insufficient differentiation by case complexity, inadequate consideration of personnel and material costs, and the current restriction of hybrid DRGs to one-day cases, which limits their applicability to more complex procedures. Experts therefore recommend a continuous, empirically based recalibration of hybrid DRG reimbursement levels [14]. The Government Commission views hybrid DRGs as a key instrument for accelerating the shift toward outpatient care and calls for their rapid further development [2].

#### Regional psychiatry budgets (§64b SGB V)

Regional psychiatry budgets represent an innovative financing mechanism that enables psychiatric instituitons to provide care flexibly across all treatment settings – home treatment, outpatient visits, day clinic, and full inpatient care – based entirely on individual patient needs. Currently, 22 clinics in nine federal states operate under this funding model, which is jointly agreed between payors and healthcare providers [15]. The model allows psychiatric hospitals to focus on continuity and therapeutic relationships rather than bed occupancy or case numbers. Evidence from the pioneering site in Itzehoe demonstrates substantial benefits: the average length of inpatient stays has been reduced by half, treatment quality has remained stable, and overall care costs have not increased over 17 years [16], [17]. A recent meta-analysis using comprehensive secondary data further confirms these positive effects and highlights the potential of regional budgets to transform psychiatric care [18]. The Government Commission notes, however, that most current §64b model projects do not yet include participation of the office-based physician sector (Kassenärztliche Vereinigungen) and recommends developing long-term concepts that can be extended to all medical specialties. A particular strength of regional budgets is their strong incentive for preventive action within the region, including outreach measures [2].

#### Telemedicine approaches in regional care: The regional telepaediatric network (RTP-NET)

Demographic changes and declining birth rates have led to a shortage of paediatric care in rural regions of Mecklenburg–Western Pomerania and northern Brandenburg. Smaller hospitals increasingly struggle to maintain paediatric units, and many families face long travel distances to the next specialist practice. To address this gap, the regional telepaediatric network (RTP-NET) was established in 2020 [19]. With 13 participating hospitals, the network offers telemedical triage, specialist consultations, virtual on-call services, and video consultations with patients and families. These digital functionalities enable faster clinical decision-making, improved access to specialist expertise, and more efficient use of regional resources. RTP-NET demonstrates how telemedicine can strengthen care structures in underserved areas, reduce avoidable transfers, and enhance the overall quality of paediatric care.

#### Best practice in surgery: Collaboration between medical care centres (MVZs) and university hospitals

Successful cooperation between medical care centres (MVZs) and university hospitals can enhance the quality of patient care and strengthen postgraduate medical training. This is illustrated by best-practice examples such as the collaboration between MVZ Chirurgie Kiel and the Clinic for Orthopaedics and Trauma Surgery at the University Medical Center Schleswig-Holstein (UKSH), which have established a joint training model. Both institutions are responding to the fact that an increasing proportion of clinical skills required in specialist training are now acquired exclusively in the outpatient sector. Overcoming longstanding biases and a lack of familiarity between sectors is therefore essential. Targeted training and information programmes can foster exchange and facilitate collaboration – for example, through jointly developed curricula for residency programs. Transparent and open communication further helps to prevent misunderstandings and build trust. Regular meetings and workshops provide a platform for sharing experiences and developing joint solutions, including standard operating procedures. Involving the relevant regional Medical Associations and Associations of Statutory Health Insurance Physicians is considered important to ensure broad acceptance and support. Cooperation between MVZs and university hospitals thus represents a promising pathway for addressing the challenges of modern healthcare. A shared vision and a collaborative approach are key prerequisites for success.

## 4 Discussion

Cross-sectoral care constitutes a central approach to improving the efficiency and quality of the German healthcare system. It addresses the challenges arising from the pronounced separation of care sectors, which leads to information loss, inefficient processes, and ultimately suboptimal patient care. Despite numerous reform efforts and initiatives to date, progress in real life remains limited. 

The planned hospital reform in Germany is regarded by many as an important step towards strengthening cross-sectoral care. However, its success will depend greatly on its concrete implementation. The reform aims to address structural problems within the healthcare system. A shift towards quality- and needs-oriented remuneration is intended to eliminate misguided incentives that have previously contributed to overtreatment or economic pressure on hospitals. 

Digitisation is another critical lever for improving cross-sectoral care. At present, however, substantial deficits remain in its implementation. Different IT systems and limited interoperability between outpatient and inpatient facilities still prevent the efficient exchange of patient data. Digitisation therefore needs to be pursued rigorously in order to establish a foundation for cross-sectoral care. The electronic patient record (ePA) ist a major step. 

A central barrier to cross-sectoral care lies in the differing remuneration systems across sectors. The introduction of hybrid DRGs represents a promising step towards integrating outpatient and inpatient services. However, their current scope of application remains limited. Further development is urgently needed to allow for a more differentiated consideration of treatment complexity as well as personnel and material costs. Regional and global budgets could offer more comprehensive solutions. (In Switzerland, a reform of the statutory health insurance system that will introduce a uniform financing in 2028–2032 has been enacted by popular vote on 24 November 2024 [20].) The regional level plays a pivotal role in the implementation of cross-sectoral care, as varying demographic and infrastructural conditions require regionally adapted solutions. 

Cross-sectoral care also requires cross-sectoral education and postgraduate training of healthcare professionals, for example within the framework of structured training networks. Best-practice examples illustrate how cross-sectoral approaches can be successfully implemented. These models highlight the importance of interdisciplinary and interprofessional collaboration. In addition, the role of nursing within interprofessional collaboration should be more clearly defined, as nursing professionals can substantially support and positively influence intersectoral processes in the direction of process optimisation. 

Health services research is essential for the planning, implementation, and optimisation of cross-sectoral care. It helps to understand and improve the complex interactions between different actors and sectors. This applies to needs assessment and planning and includes the examination of regional differences as well as the estimation of future care needs, such as risk stratification models in the context of demographic change or shifting disease patterns. The evaluation of innovative care models in terms of clinical efficiency and cost-effectiveness, acceptance, and implementation also represents a core domain of health services research [21]. Patient-related aspects are of particular relevance. PROMs (Patient-Reported Outcome Measures) and PREMs (Patient-Reported Experience Measures) are used to systematically capture the patient perspective and represent central metrics of patient-centred care [22]. Research designs include prospective, cluster-randomised intervention studies, stepped-wedge designs, prospective studies with non-randomised control groups, and a posteriori matching. Fully randomised designs are not feasible for all research questions [22]. 

Cross-sectoral monitoring also plays a key role. It refers to the systematic collection, analysis, and monitoring of healthcare processes and outcomes. Its purpose is to create transparency, ensure quality, and identify areas for improvement. Monitoring aims to detect gaps in care, inefficient processes, or unequal access conditions. It provides the basis for evidence-based governance and data-driven decisions regarding the further development of care structures. This requires robust indicators and feedback systems for providers in order to initiate improvements. A cross-sectoral basic indicator set for healthcare provision in rural areas is currently under development [23].

To move consistently towards integrated care, decisive measures are necessary. These include promoting comprehensive and consistent digitisation at all levels, restructuring remuneration models, and strengthening regional networks. At the same time, legal and structural barriers must be dismantled, and successful models need to be scaled systematically. Health services research is indispensable for all phases of planning, implementing, and optimising cross-sectoral care. Cross-sectoral monitoring supports evidence-based governance and enables data-driven decisions for the further development of care structures.

### Recommendations of the AWMF

The AWMF recommends the consistent further development and establishment of cross-sectoral, evidence-based care to improve the efficiency and quality of the German healthcare system. Close cooperation between health policy, scientific medicine, and health services research is essential. The reform approaches initiated between 2021 and 2025 should be further advanced with the active involvement of the scientific medical societies.

Specific proposals resulting from the current article are summarized in Table 4 (Tab. 4). The lack of harmonisation in remuneration systems between inpatient and outpatient services is a major stumbling block for implementation of the multitude of existing integrated care models. Regional, population-based planning of healthcare networks may offer a solution. Digitisation of the healthcare system can support this, provided it leads to smooth information exchange across healthcare sectors. Shared decision-making by patients and physicians regarding the appropriate form of care within an evidence-based set of options should be a guiding principle. The AWMF further emphasizes the need for integrated education and postgraduate training, particularly within structured residency networks. 

## Notes

### Acknowledgements

We would like to thank the external speakers and panelists at the Berlin Forum: Andreas Bechdolf, Tom Bschor, Jean-François Chenot, Siiri Doka, Gerald Gaß, Wolfgang Hoffmann, Peter Hüttl, Anita Jagota, Susanne Johna, Peter Kalbe, Frederik Schlottmann, Ralf Schmitz, Nobert Suttorp, Neeltje van den Berg, Susanne Weinbrenner and all participants of the resulting discussions for their intellectual stimuli. 

### Competing interests

The authors declare that they have no competing interests.

## Figures and Tables

**Table 1:**
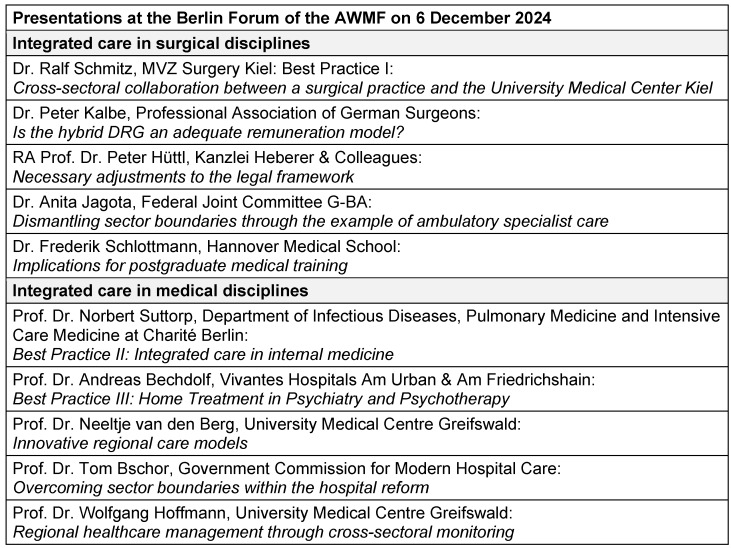
Presentations at the AWMF Berlin Forum on 6 December 2024 [26]

**Table 2:**
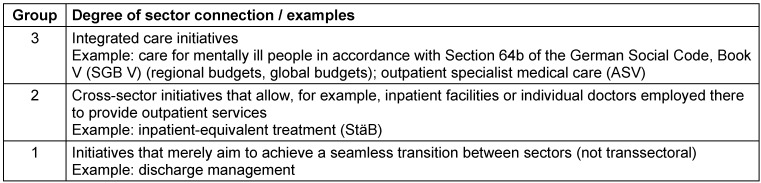
Systematization of initiatives for integrated care according to their degree of sector connection (cf. [2])

**Table 3:**
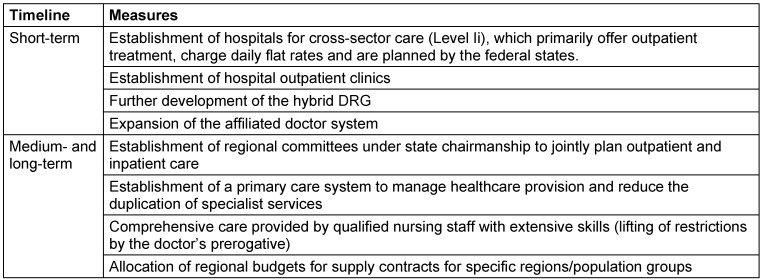
Summary of recommendations for overcoming sector boundaries in the German healthcare system by the Government Commission for Modern, Needs-Based Hospital Care (2024) [2]

**Table 4:**
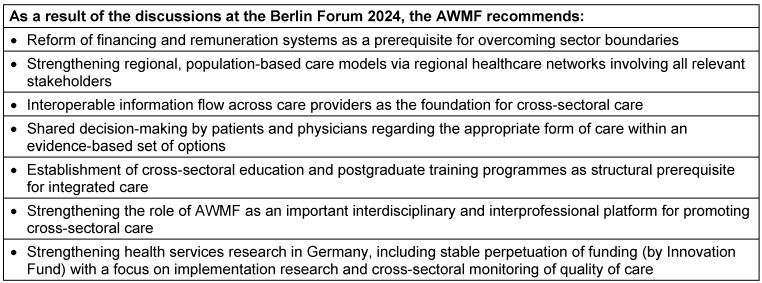
AWMF recommendations for implementation of cross-sectoral care in Germany
